# Early warning indicators for mesophilic anaerobic digestion of corn stalk: a combined experimental and simulation approach

**DOI:** 10.1186/s13068-019-1442-7

**Published:** 2019-05-03

**Authors:** Yiran Wu, Adam Kovalovszki, Jiahao Pan, Cong Lin, Hongbin Liu, Na Duan, Irini Angelidaki

**Affiliations:** 1 College of Water Resources and Civil Engineering, China Agricultural University, Beijing, 100083 China; 2 Department of Environmental Engineering, Technical University of Denmark, 2800 Kgs. Lyngby, Denmark; 3 Key Laboratory of Nonpoint Source Pollution Control, Ministry of Agriculture/Institute of Agricultural Resources and Regional Planning, Chinese Academy of Agricultural Sciences, Beijing, 100081 China

**Keywords:** Corn stalk, Anaerobic digestion, Early warning, BioModel

## Abstract

**Background:**

Monitoring and providing early warning are essential operations in the anaerobic digestion (AD) process. However, there are still several challenges for identifying the early warning indicators and their thresholds. One particular challenge is that proposed strategies are only valid under certain conditions. Another is the feasibility and universality of the detailed threshold values obtained from different AD systems. In this article, we report a novel strategy for identifying early warning indicators and defining threshold values via a combined experimental and simulation approach.

**Results:**

The AD of corn stalk (CS) was conducted using mesophilic, completely stirred anaerobic reactors. Two overload modes (organic and hydraulic) and overload types (sudden and gradual) were applied in order to identify early warning indicators of the process and determine their threshold values. To verify the selection of experimental indicators, a combined experimental and simulation approach was adopted, using a modified anaerobic bioconversion mathematical model (BioModel). Results revealed that the model simulations agreed well with the experimental data. Furthermore, the ratio of intermediate alkalinity to bicarbonate alkalinity (IA/BA) and volatile fatty acids (VFAs) were selected as the most potent early warning indicators, with warning times of 7 days and 5–8 days, respectively. In addition, IA, BA, and VFA/BA were identified as potential auxiliary indicators for diagnosing imbalances in the AD system. The relative variations for indicators based on that of steady state were observed instead of the absolute threshold values, which make the early warning more feasible and universal.

**Conclusion:**

The strategy of a combined approach presented that the model is promising tool for selecting and monitoring early warning indicators in various corn stalk AD scenarios. This study may offer insight into industrial application of early warning in AD system with mathematical model.

**Electronic supplementary material:**

The online version of this article (10.1186/s13068-019-1442-7) contains supplementary material, which is available to authorized users.

## Background

Anaerobic digestion (AD), as an efficient technology for organic waste treatment, has been widely adopted worldwide [1]. Among others, straw has a great potential to serve as a feedstock for anaerobic methane (CH_4_) production, due to its abundance and suitable bioconversion characteristics [2]. In China, straws are produced at a high annual rate of approximately 1 billion metric tons [3], and about 30% of those were underutilized [3, 4]. In addition, the methanogenic potential of some major straws is 2.86–3.78 × 10^5^ Nm^3^ CH_4_/kg VS [5]. However, straw is consisting of cellulose, hemicellulose, and lignin, which is a typical high carbon-to-nitrogen (C/N) substrate [2, 6]. And excessive volatile fatty acids (VFAs), which are intermediates of AD, may be produced when feedstock overloading occurs, especially with high C/N (e.g., > 30) [2, 5, 7]. Besides, the complex structure of lignocellulose is difficult for microbial cellulolytic enzymes to access, limiting degradation [8]. Therefore, some previous studies reported that the moderate organic loading rates (OLRs) were very vital to avoid system acidification. The thermophilic AD process of straw can only be stably operated under relatively low organic loading rates (OLRs) and below 2 kg VS/(m^3^ day) [9]. Meanwhile, Li et al. [10] suggested that the mesophilic anaerobic co-digestion of rice stalk with cow manure should be operated at an OLR of 3–6 kg VS/(m^3^ day). Ward et al. [11] found that biogas projects using straw as substrate also tend to be controlled at suboptimal OLRs to prevent process inhibition.

Besides optimal operational parameters like OLR, reliable early warning and regulation systems are also favorable for AD process. Previously, many studies have been carried out to explore feasible warning indicators in different AD systems. Some examples for several indicators proposed include VFA concentrations, alkalinity, biogas composition, specific intermediate metabolite (like glycerol, aromatic compound, etc.) concentrations, microbial community composition, and enzyme activity [12–17]. In addition, some coupled indicators, such as the ratio between intermediate and partial alkalinities or VFA concentrations and bicarbonate alkalinity (BA), showed better performance than individual indicators [18]. However, when comparing the results of previous studies, it must be pointed out that the proposed strategies are only valid under certain conditions, as some parameters may have different sensitivities to environmental fluctuations in different AD systems. For instance, Castellano et al. [19] suggested that hydrogen (H_2_) concentrations have a high discriminatory ability for process state identification. On the contrary, Kleyböcker et al. [20] showed that H_2_ partial pressure was not an ideal indicator, because of its unstable responses under organic overload conditions in an AD system treating rapeseed oil. In addition, the indicator threshold values might vary in AD systems with different substrates and operating conditions. In a specific case, Pullammanappallil et al. [21] suggested that the critical value of propionic acid was 2750 mg/L, while Holm-Nielsen et al. [22] found it to be 1500 mg/L. Conversely, propionic acid did not show early warning in some trials [20]. Consequently, finding effective indicators and rational threshold values for early warning and inhibition diagnosis is a challenge when working with AD systems.

Compared with traditional early warning methods, which only monitor process indicators by chemical pathway, modeling the AD process can provide a flexible and rapid solution for comparing and evaluating large numbers of such indicators, and it provides the possibility of automated warning for industrial applications. Mathematical models have long been used for the simulation of various AD scenarios, with several computer-aided implementations in existence [23, 24]. Unlike simple models that are mainly used for calculating theoretical biogas and CH_4_ yields, complex bioconversion models can be used to generate insights about process kinetics, microbial growth inhibition, substrate conversion, and product generation rates, to mention a few of their functionalities. In addition, these models can also handle extensive amounts of numerical data and provide qualitative or quantitative comparisons between the measured and simulated datasets. Hence, using these tools for the evaluation of early warning indicators appears to be a promising approach.

The aims of the present study were therefore to (1) evaluate the reaction of various process parameters during AD process of corn stalk fed to continuously stirred tank reactors under different overload modes (organic or hydraulic retention time (HRT) overload; and gradual or sudden overload); (2) compare simulation using a proven bioconversion model and experimental results at the same operational conditions; (3) identify and evaluate response parameters that are sufficiently sensitive to environmental disturbances; and (4) define threshold values for the sensitive indicators identified in (3), for the different overload conditions tested.

## Results and discussion

### Digester performance

#### Gaseous parameters

**Fig. 1:**
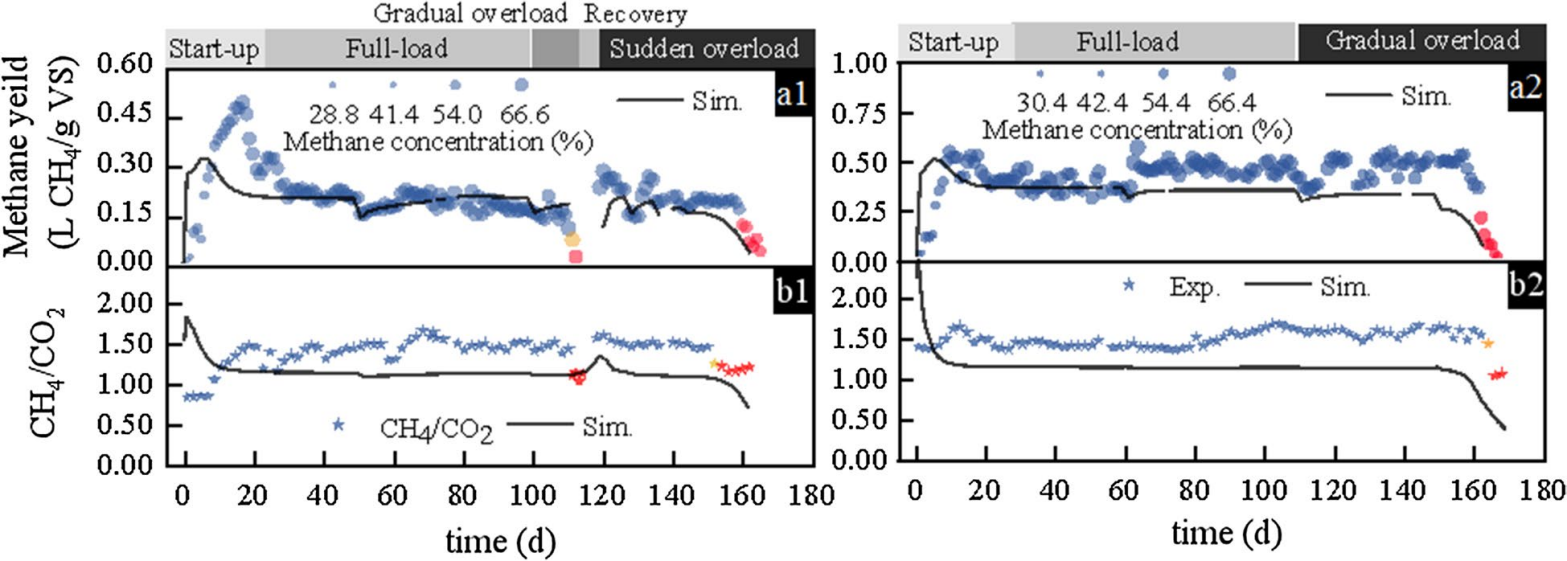
A comparison of experimental and simulated CH_4_ yields (**a1**, **a2**) and CH_4_/CO_2_ ratios (**b1**, **b2**) related to the laboratory experiments. Data presented in subplots **a1** and **b1** represent R1, while those in subplots **a2** and **b2** represent R2

The CH_4_ yield and content of the two reactors were seen to have stabilized in the full-load phase, at 0.20 L CH_4_/g VS (62.20% of CH_4_) on average with OLR of 1.50–2.24 g VS/(L day) in R1 and at 0.20 L CH_4_/g VS (58.73% of CH_4_) with OLR between 1.87 and 2.24 g VS/(L day) in R2. During the gradual organic overload phase in R1 (day 101–113), both the CH_4_ yield and CH_4_ content decreased significantly to 0.02 L CH_4_/g VS and 51.76% on day 113. Regarding the sudden overload phase of R1, the CH_4_ yield showed a stepwise rise with elevated OLR, up to 0.37 L CH_4_/g VS on day 155 with OLR at 3.37 g VS/(L day), and then sharply decreased to 0.12 L CH_4_/g VS (day 161). With respect to the gradual HRT overload phase of R2, the CH_4_ yield showed an acidification response under OLR of 2.81–3.74 g VS/(L day). Subsequently, CH_4_ production increased slowly and then decreased sharply after day 161. For each overload condition, the CH_4_ content decreased slightly in the beginning and then returned to steady-state level (Fig. 1a1, a2). This result was consistent with a previous study, where the CH_4_ content did not decrease significantly, as long as the pH was higher than 5.5 [1]; moreover, it reported that raising OLR did not result in a significant change in CH_4_ content. However, a clear shift in populations of archaea from acetotrophic to hydrogenotrophic methanogenesis may have occurred [25].

**Fig. 2:**
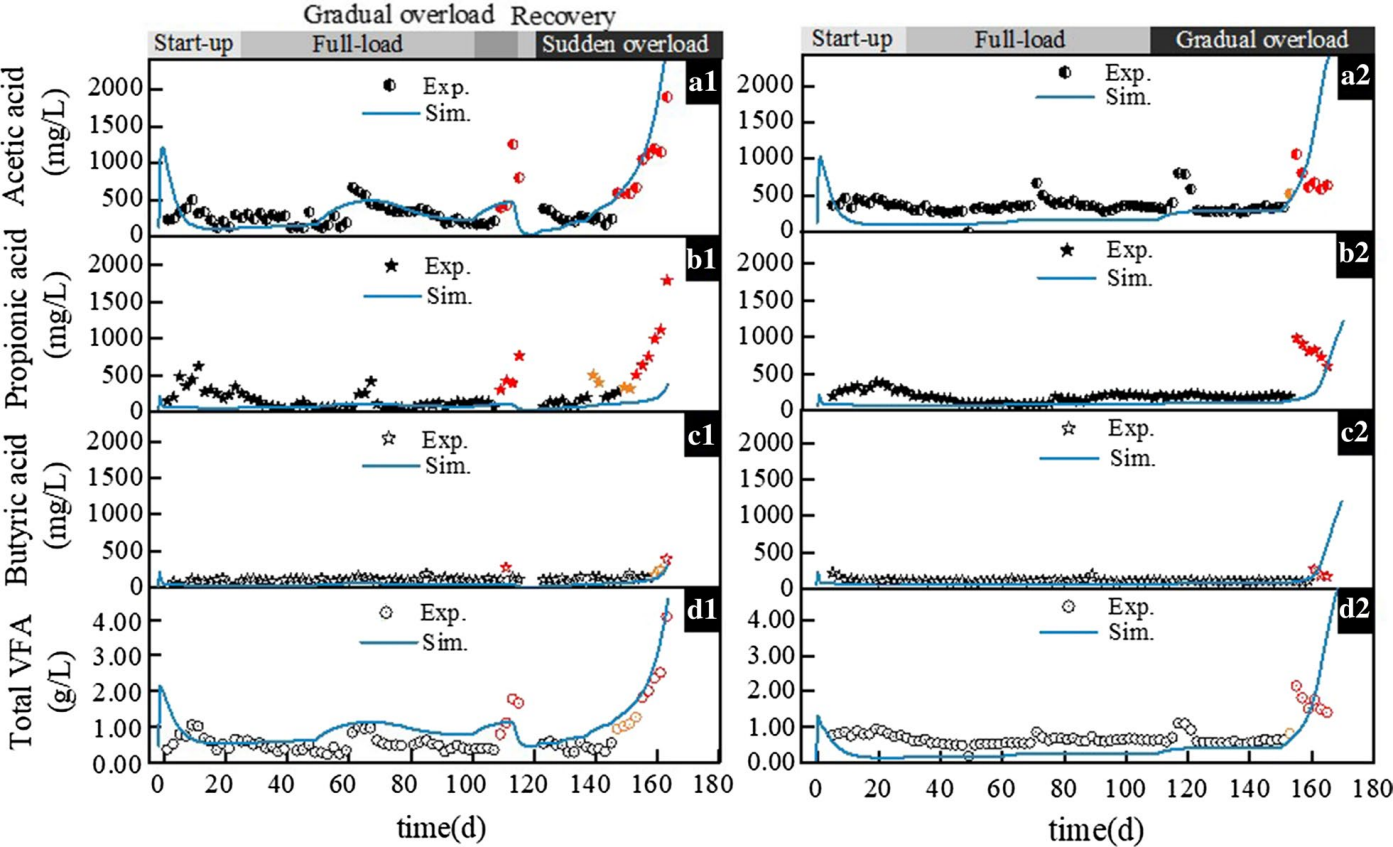
A comparison of experimental and simulated acetic acid (**a1**, **a2**), propionic acid (**b1**, **b2**), butyric acid (**c1**, **c2**), and total VFA (**d1**, **d2**) concentrations related to the laboratory experiments. Data presented in subplots **a1**, **b1**, **c1**, and **d1** represent R1, and those in subplots **a2**, **b2**, **c2**, and **d2** represent R2

On the other hand, solids evidently accumulated in the reactor, probably due to straw floating and inadequate stirring, which is approximately 5–6% in the last phase of the experiment (Additional file 1: Fig. S1). Solids accumulated could lead to gas–liquid phase transfer delay and oversaturation of CH_4_ in the liquid phase [26]. Thereby, above gaseous parameters were relatively insensitive. Meanwhile, the ratio of methane and carbon dioxide (CH_4_/CO_2_) showed more intense response to perturbations (Figs. 1b and 2b), where sudden drops were observed on days 112 and 161 in R1, and on day 161 in R2. Further, it realized a remarkable early warning potential in comparison with CH_4_ yield (8 days earlier) under sudden overload, but barely provided any early warning under gradual overload. Previously, H_2_ concentration was also suggested as a useful variable for detection of disturbances in both carbohydrate- and protein-based wastewaters [27]. However, in the current study, H_2_ was only detected in the overload stage on days 112 and 155 in R1, and on day 159 in R2. In addition, these fluctuations were faint and short (1–2 days), with the maximum H_2_ content of 0.17% being reached in the sudden overload phase of R1. It is speculated that the pH and total solid (TS) concentration of the effluent can affect the discrimination of H_2_.

#### VFA parameters

As far as the full-load phase is concerned, the total VFA concentrations of the two reactors were found stable, with average values of 0.49 g/L (R1) and 0.60 g/L (R2), respectively. However, the total VFA concentration increased rapidly during the overload phase. Acetic acid was the most abundant acid, accounting for approximately 40% of the total VFA, hence it dominates total VFA concentration changes. Its sharp increase occurred on day 109 (from 0.21 to 0.39 g/L) and day 147 (from 0.23 to 0.59 g/L) in R1, and on day 155 (from 0.34 to 0.52 g/L) in R2. The sudden rise of total VFA was also occurred on day 109 (from 0.37 to 0.80 g/L) and day 147 (from 0.56 to 0.95 g/L) in R1, and on day 155 (from 0.82 to 2.15 g/L) in R2. The quick accumulation of acetic acid indicated an imbalance between the acid-forming phase and methane-forming phase of the digestion process, which was in line with the changes seen in CH_4_ yield and CH_4_/CO_2_ (Fig. 1). Propionic acid concentration was fairly stable during the full-load phase, especially in R2 (Fig. 2c1, c2). Its sharp increase was observed on day 109 (from 0.06 to 0.30 g/L) and day 153 (from 0.32 to 0.50 g/L) in R1, and on day 155 (from 0.18 to 0.98 g/L) in R2. It is noticeable that in R2, the sharp increases of VFAs concentration were always followed by the gradual returning to its steady-state concentration, and propionic acid showed the slowest recovery (Fig. 2), which is in agreement with a previous study [22], but different to the finding of Boe et al. [28] stating that acetate revealed recovery while propionate was persistent. In the case of methanogenic populations, possible adaptation to the higher VFA concentrations may have occurred, which was probably the reason for reduced acidification [29]. What’s more, a latest research reported that there is a kind of methanogen could uptake acetate but also propionate directly, which might be one of the reasons as well [30]. The findings about slow propionic acid reduction were also supported by the findings from Ahring et al. [31], who showed that propionate acid degraders are the slowest growing and most sensitive VFA-degrading microorganisms in the AD process.

### pH, alkalinity, and VFA/BA

During the full-load period of the experiment, pH values ranged from 6.77 to 6.90 (R1) and 6.67 to 6.83 (R2), respectively (Additional file 1: Fig. S2). In overload period, the pH value decreased below 6.4 on day 112 (R1-gradual overload), on day 159 (R1-sudden overload), and on day 160 (R2-gradual overload), respectively, which value we defined as limit for process failure.

**Fig. 3:**
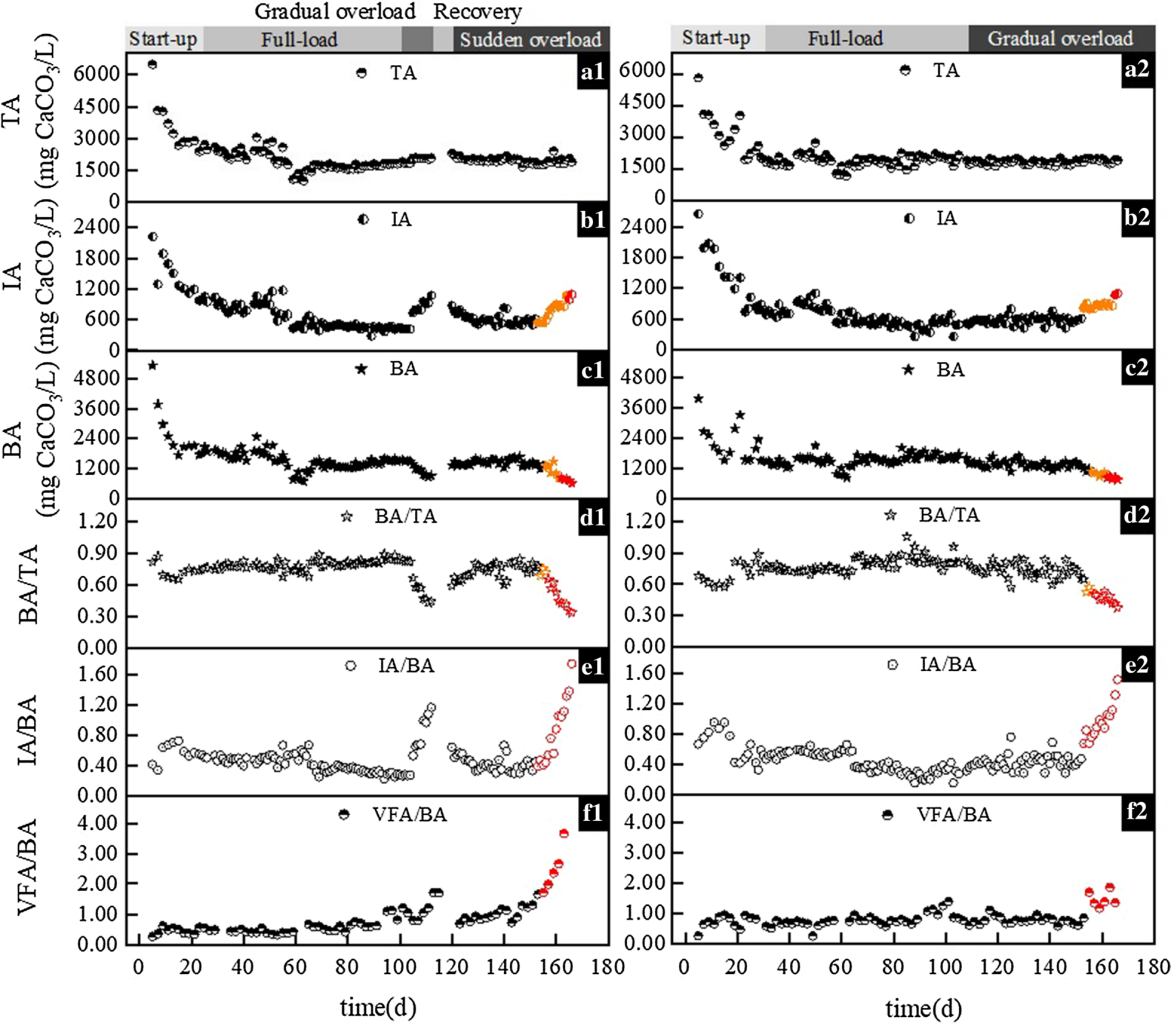
A comparison of experimental and simulated total alkalinities (**a1**, **a2**), intermediate alkalinities (**b1**, **b2**), bicarbonate alkalinities (**c1**, **c2**), BA/TA (**d1**, **d2**), IA/BA (**e1**, **e2**), and VFA/BA (**f1**, **f2**) ratios related to the laboratory experiments. Data presented in subplots **a1**, **b1**, **c1**, **d1**, **e1**, and **f1** represent R1, while those in subplots **a2**, **b2**, **c2**, **d2**, **e2**, and **f2** represent R2

High total alkalinity (TA) (about 6000 g CaCO_3_/L) was detected in both reactors at the beginning, due to the high alkalinity contained in the inoculum originating from the full-scale biogas plant. During the experiment, TA was maintained at an average of 1814.3 mg CaCO_3_/L (R1) and 1871.9 mg CaCO_3_/L (R2) (Fig. 3a1, a2). The result was also in accordance with a previous study, in that TA could remain stable until the pH fell below 4.3 as a consequence of high VFA concentration [32]. BA suddenly dropped on days 109 and 158 in R1, and day 154 in R2 (Fig. 3c1, c2). Conversely, intermediate alkalinity (IA) suddenly rose on days 105 and 157 in R1, and day 153 in R2 (Fig. 3b1, b2). Although both indicators showed fluctuation under overload conditions, IA proved to be more sensitive, since it got out of balance earlier.

From another perspective, coupled indexes appeared to be more sensitive than single indicators, thus their distinct fluctuations made the identification of acidification response easier. BA/TA decreased sharply on days 105 and 158 in R1, and on day 154 in R2 (Fig. 3d1, d2). Conversely, IA/BA showed a sharp increase on days 105 (from 0.28 to 0.53) and 157 (from 0.44 to 0.54) in R1, and day 153 (from 0.48 to 0.68) in R2 (Fig. 3e1, e2). Unfortunately, all alkalinity indicators showed obvious signs of response delay under sudden overload. It is similar to the report of Li et al. [33] where the warning times of IA/PA (where PA is the partial alkalinity and is analogous to BA) and BA/TA were shortened by 6 days and 2 days, respectively. Ahring et al. [12] also reported that most indicators were suitable for detecting gradual overloads, but were too slow to respond under sudden overload. Significant increase of VFA/BA was found on days 113 and 151 in R1, and day 155 in R2 (Fig. 3f1, f2), and was confirmed by the work of Li et al. [33], who also found VFA/BA increase due to higher OLR in mesophilic AD of vegetable waste.

### Simulation results using the BioModel

The results of the experimental reactor operation simulations are presented in terms of CH_4_ yield and CH_4_/CO_2_ (Fig. 1), and individual and total VFA concentrations (Fig. 2). From a qualitative point of view and by looking at the fits between experimental and simulated data curves, it appears that the model was mostly successful in capturing the overall gas and VFA production trends of the two experiments with high accuracy. At the same time, by evaluating the goodness of these fits using statistical measures, relative root mean squared error (rRMSE) and mean absolute percentage error (MAPE) values provide a more detailed overview of simulation accuracy (Table 1). By comparing the visual and statistical measures, biogas and CH_4_ yield, along with butyric and acetic acid concentration simulations appear to be the most accurate, with the lowest rRMSE and MAPE only slightly above the feasible range (Table 1, values for “Day 0–165”). This deviance is due to a few sections of the dataset, where the simulation-measurement fit was not satisfactory. For example, the gas yield and VFA concentration levels during the reactor startup periods were not matched in the simulations. However, reactor startup periods inherently involve significant stochasticity and, depending on the substrates and reactor history, potential microbial growth lag [34], which are hard to simulate with fixed kinetic models. Deeming this period irrelevant from the perspective of early warning indication and excluding it from the statistical evaluation, rRMSE and MAPE values of CH_4_ yield (Table 1, values for “Day 30–165”) have improved significantly. On the other hand, VFA simulations showed varying rates of change, with those of R1 being more positive than R2. A more important difference between the measured and simulated values was, on the other hand, seen in R1, around days 110–120. Here, the drop in CH_4_ yield and the consecutive process failure were slower in the simulation results compared to the laboratory experience, by approximately 2 days. At the same time, the drop was preceded by a slight increase in the respective yields, potentially owing to an initial positive response of simulated acetoclastic methanogens to an increased OLR. This means that in the model, the immediate response of microbial groups to an increase in substrate availability is a proportional increase in their productivity, which is in most cases followed by their negative response to the gradual accumulation of certain inhibitory compounds. In the case of acetoclastic methanogens, model inhibition was assumed by free ammonia and the saturation of volatile fatty acids, of which the latter was seen both experimentally and during the simulations. However, as the levels of acid saturation were significantly lower in the model simulations than in the physical reactors, inhibition during the simulations appeared to take effect slower than seen experimentally. Nevertheless, the simulations for both R1 and R2 were in general found to be in good agreement with experimental data, therefore they could be used for comparing the experimentally defined early warning indicators with their simulated counterparts. In the short term, this provided an additional method for verifying the quality of indicator selection. In addition, the long-term benefits of such simulations lie in their rapid generality and continuous interpretability: both contributing to the reduction of necessary analytic measurements and process data density. Thus, by means of simulated early warning indicators, monitoring, and forecasting, the fate of the experimental processes could be improved significantly.

**Table 1:** rRMSE and MAPE values for goodness-of-fit analyses

Process variable	R1				R2			
	rRMSE		MAPE (%)		rRMSE		MAPE (%)	
	Day 0–165	Day 30–165	Day 0–165	Day 30–165	Day 0–165	Day 30–165	Day 0–165	Day 30–165
CH_4_ yield	0.41	0.23	57.58	19.78	0.28	0.12	39.79	11.30
CH_4_/CO_2_	2.44	2.79	31.21	31.38	1.33	1.24	25.72	25.30
Acetic acid conc.	0.73	0.64	52.20	43.14	0.85	0.87	54.15	52.13
Propionic acid conc.	1.21	1.30	79.02	78.46	0.94	0.95	64.93	61.18
Butyric acid conc.	0.20	0.20	61.81	60.36	0.24	0.23	70.44	68.74
Total VFA conc.	0.96	0.80	40.85	32.16	1.25	1.27	53.12	50.12

### Early warning indicators and threshold values

#### The procedure of screening early warning indicators

Experimental and simulated data were used to screen potentially optimal warning indicators, based on the proposition of three important and mostly qualitative criteria. Firstly, optimal indicators had to show high sensitivity to changes, in the sense that there was enough warning time between an indicator’s response point and process failure. Stable acidification response was another vital aspect, as excessive indicator sensitivity to acids may have led to false assumptions. Finally, the low cost of monitoring is essential in practice [20, 35], therefore indicators had to be measured economically.

Based on the above selection criteria, reference points for reactor failure are needed for measuring warning time of different parameters. In the current study, these points of failure were declared when at least one of two events happened. The first one concerned the reduction of reactor pH to less than 6.4, which is well below the optimal pH range commonly reported for methanogenic archaea [36]. Meanwhile, the other event was the significant reduction of CH_4_ yield (relative standard deviation (RSD) > 20%). pH and CH_4_ yield are the most intuitive and easily measured indicators, but relatively insensitive for process destabilization, therefore, their changes were also recommended as the symbol of process failure in previous study [37].

In order to quantify the changes of different indicators at the points of failure, their variation amplitudes were calculated. These amplitudes were expressed on a percentage basis and relative to their steady-state values. Specifically, by comparing the variation amplitude of abrupt changes throughout the different overload modes and types applied in the experimental reactors, maximum and minimum values of each indicator were defined.

#### Screening early warning indicators

According to the experimental data, days 112 and 159 were identified as reference points for measuring the early warning times of different indicators under gradual and sudden overload conditions in R1, and this reference point was day 160 for R2.

The abrupt change dates and values, and warning times of different indicators are shown in Tables 2 and 3. Almost all the parameters have performed certain response to the overload shock. Overall, gas phase indicators provided a delayed response in comparison with liquid phase indicators, only CH_4_/CO_2_ have 6 days warning time under sudden overload. This could probably be due to the properties of corn stalk (CS), given the floating problem caused by low specific gravity can lead to stirring issues and eventually delays in mass transfer from the liquid to the gas phase [38, 39].

**Table 2:** The early warning indicators of R1

Reactor	Parameters	Units	Date of abrupt change (day)	Date of process failure (day)	Warning time (day)	Experimental results		Simulated results	
						Abrupt change^a^	Abrupt vs. steady-state value	Abrupt change	Abrupt vs. steady-state value
R1	CH_4_/CO_2_	–	112 (153)	112 (159)	0 (6)	1.28 (1.43)	− 23.81 to − 18.48% (− 16.38 to − 11.73%)	1.07 (1.03)	− 2.49% (− 11.95 to − 1.60%)
	Acetate	g/L	109 (147)	112 (159)	3 (11)	0.39 (0.59)	+ 105.26 to 129.41% (+ 145.83%)	0.44 (0.56)	+ 27.52 to 94.71% (+ 48.06 to 234.57%)
	Propionate	g/L	109 (153)	112 (159)	3 (6)	0.3 (0.5)	+ 130.77 to 172.73% (+ 177.78 to 257.14%)	0.09 (0.12)	+ 18.46 to 101.87% (+ 101.76 to 274.62%)
	VFA	g/L	109 (147)	112 (159)	3 (12)	0.80 (0.95)	+ 50.9 to 105.13% (+ 75.93 to 150%)	0.63 (0.76)	+ 19.49 to 22.20% (+ 56.02%)
	IA	g CaCO_3_/L	105 (157)	112 (159)	7 (2)	0.79 (0.74)	+ 61.48% (+ 20.68 to 30.65%)	0.28 (0.87)	+ 18.54 to 19.68% (+ 63.22 to 180.86%)
	BA^b^	g CaCO_3_/L	107 (158)	112 (159)	5 (1)	1.21 (1.04)	− 20.43 to − 11.10% (− 23.22 to − 22.85%)	1.07 (0.70)	− 32.84% (− 27.40 to − 50.32%)
	VFA/BA	–	109 (147)	112 (159)	3 (12)	0.77 (0.68)	+ 113.89 to 196.15% (+ 183.33%)	0.59 (0.70)	− 23.06 to − 69.02% (+ 199.76%)
	BA/TA	–	105 (158)	112 (159)	7 (1)	0.67 (0.56)	− 19.28 to − 14.11% (− 30.86 to − 25.34%)	0.80 (0.43)	− 14.38% (− 51.77 to − 33.80%)
	IA/BA	–	105 (157)	112 (159)	7 (2)	0.53 (0.54)	+ 10.42 to 70.97% (+ 23.26%)	0.26 (1.06)	+ 20.06 to 26.02% (+ 86.01%)
	pH	–	–	112 (159)	–	6.24 (6.38)	–	<6.4	–
	CH_4_ yield^c^	L CH_4_/g VS	–	113 (161)	–	0.02 (0.12)	–	0.20 (0.04)	+ 47.01 to 68.95% (− 32.33 to − 13.55%)

^a^Values in the units indicated in the “Parameters” column

^b^BA in simulated results expressed as the absolute change in the sum of dissolved HCO_3_^−^ and CO_3_^2−^ over half a day, in %

^c^The values in brackets refer to the corresponding values of sudden overload. – means decrease, and +  means increase

**Table 3:** The early warning indicators of R2

Reactor	Parameters	Units	Date of abrupt change (day)	Date of process failure (day)	Warning time (day)	Experimental results		Simulated results	
						Abrupt change^a^	Abrupt vs. steady-state value	Abrupt change	Abrupt vs. steady-state value
R2	CH_4_/CO_2_	–	161	160	0	1.00	− 36.31 to − 25.48%	0.86	− 18.01 to − 21.33%
	Acetate	g/L	153	160	7	0.52	+ 57.58 to 73.33%	0.43	+ 36.43 to 303.98%
	Propionate	g/L	155	160	5	0.98	+ 390.00 to 444.44%	0.12	+ 27.64 to 206.45%
	VFA	g/L	155	160	5	2.15	+ 225.76 to 305.66%	0.80	+ 76.84 to 193.84%
	IA	g CaCO_3_/L	153	160	7	0.87	+ 37.09 to 62.18%	0.32	+ 36.66 to 138.60%
	BA**	g CaCO_3_/L	154	160	6	1.04	− 34.29 to − 22.27%	0.89	− 60.09 to − 11.46%
	VFA/BA	–	155	160	5	0.64	+ 36.17 to 88.24%	0.94	+ 106.41 to 338.75
	BA/TA	–	154	160	6	0.52	− 36.59 to − 28.77%	0.71	− 25.57 to − 13.03%
	IA/BA	–	153	160	7	0.68	+ 47.83 to 65.95%	0.34	+ 24.32 to 99.59%
	pH	–	–	160	–	6.33	–	<6.4	–
	CH_4_ yield***	L CH_4_/g VS	–	163	–	0.09	–	0.07	− 69.97 to 52.38%

^a^Values in the units indicated in the “Parameters” column

^b^BA in simulated results expressed as the absolute change in the sum of dissolved HCO_3_^−^ and CO_3_^2−^ over half a day, in %

^c^The values in brackets refer to the corresponding values of sudden overload. − means decrease, and + means increase

It is obvious that parameters’ sensitivity is influenced by the overload mode. For the gradual overload, the acidification responses of the various indicators did not show significant difference (3–7 days for individual VFA and 6–7 days for alkalinity) (Tables 2 and 3), regardless of the overload type. Compared with the gradual overload, indicators demonstrated varied acidification responses during sudden overload. More specifically, VFA-related indicators (6–12 days) had longer warning time, while alkalinity and its coupled indicators (1–2 days) had shorter warning time (Tables 2 and 3). This contradiction is probably related to the change of alkalinity. At the beginning of the AD process, the effluent taken from the full-scale plant for inoculation provided a high initial alkalinity in both reactors. Later, the gradual overload applied to R1 generated a large amount of VFA, most of which was consumed by alkalinity that delayed VFA accumulation. By contrast, as the buffer capacity of the AD system declined before the second (sudden) overload phase, VFA accumulated rapidly and showed a longer warning time.

Besides early warning time, stability and measurability of indicators are also important for monitoring biogas plants [19]. For example, IA and IA/BA, as well as BA and BA/TA, showed similar warning times in the two reactors (Tables 2 and 3). However, the average RSD values of these indicators during the full-load phase were 9.57 and 11.59 for IA, 8.23 and 10.31 for IA/BA, 6.26 and 6.94 for BA, and 2.83 and 3.45 for BA/TA, in R1 and R2, respectively. The RSD values of coupled indicators are relatively smaller, which indicates that the coupled indicators are more consistent before overload shock and implies less misjudgment as well. Furthermore, AD is an interrelated process, and the relationship between certain process variables might be the reason for coupled indicators showing better stability than individual ones. Although Li et al. [18] also proposed using coupled indicators to achieve early warning, present study showed that total VFA, acetic, and propionic acids can provide similar warning times in the reactors (Tables 2 and 3). Nevertheless, total VFA can be determined relatively easier than individual VFA in biogas plants [40], making total VFA a more widely acceptable variable for early warning indication. Fortunately, due to the development of detection technology, total VFA and alkalinity can both be monitored online by transducers, titration, or infrared spectroscopy, as well as via online sampling and gas chromatography [41–44].

In addition to the experiment-based identification of indicator variables with the highest early warning potential, results of the simulations were used to calculate the same variables numerically and evaluate their warning efficiency in comparison to the empirical values. This approach was unlike any previous work on the topic that the authors reviewed [28, 45–49]. Up until now, early warning indicators were selected mostly based on the offline or online monitoring of different values of VFA, pH, alkalinity, biogas fractions, trace elements, and their various combinations, although, in one instance, stable carbon isotopes of CH_4_ were also named as potential indicators of process imbalance [50]. These indicator values could then be either evaluated comparatively, or used as inputs to sophisticated control systems. Despite their usually good early warning indicator potential, so far the majority of these solutions remained inapplicable by the industry, mainly due to their limited scope or significant costs involved [51]. Therefore, present simulation-based system could offer a competitive solution for the identification and monitoring of early warning indicators, through its validation by relevant experimental data, application flexibility, customizability at scale, and relatively low price point, when compared to laboratory-intensive processes.

**Fig. 4:**
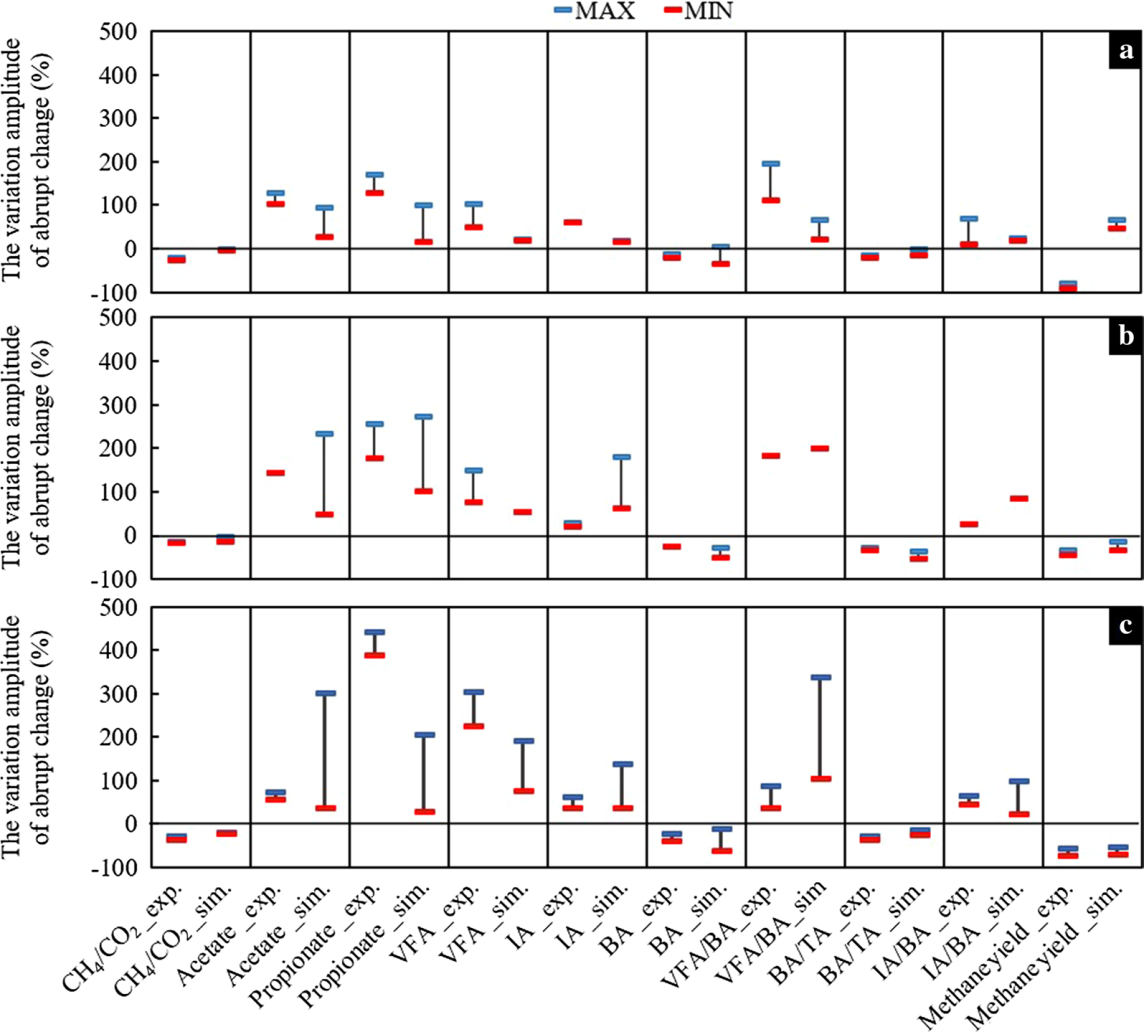
A comparison of experimental and simulated early warning indicator values in R1 during gradual overload (**a**) and sudden overload (**b**), and in R2 during gradual overload (**c**). Negative values indicate a decrease, while positive values indicate an increase in the value of the respective indicators, at the points of abrupt changes and relative to steady-state values

The experimental and simulated variables were compared separately for reactors R1 and R2 and in the gradual (Rg) and sudden overload (Rs) modes, thus creating three distinct indicator groups of interest, referred to as R1 g, R1 s, and R2 g from here onwards. In order to provide a reasonable basis for indicator comparison, reference values for identifying the early warning time in the simulations were chosen according to the experimental points defined earlier. Figure 4 shows a comparison of the experimental and simulated indicators for the three indicator groups described above. For each group and individual indicator, the orange bars enclosed with red (lower) and blue (higher) horizontal lines represent the ranges of percentagewise differences calculated between the reference (critical) indicator values and their highest values measured (*exp)* or simulated (*sim*) during the different steady-state periods (Tables 2 and 3). As an example, for the indicator group R2 g (Fig. 4c1, c2), the reference value of the experimental propionate indicator (*Propionate_exp*) was found to be 0.98 g/L on day 155; then during the five steady-state periods identified from the gradual mode operational data of R2, the highest experimental values measured were given as 0.20, 0.23, 0.22, 0.20, and 0.19 g/L, representing, respectively, 390, 326, 345, 390, and 416% changes between their values and the reference value of 0.98 g/L; finally, selecting the lowest (326%) and the highest (416%) changes, the minimum and maximum boundaries of the bar belonging to R2 g *Propionate_exp* (Fig. 4c1, c2) were given. The ranges for the simulated propionate indicator (*Propionate_sim*), along with all the other indicators were calculated in an identical manner. In cases where the minimum and maximum indicator values were equal, average indicator values were used for the calculation (see standalone red horizontal lines).

Figure 4 shows that the experimental and simulated difference ranges agreed well for some indicators, while for others they were significantly different. Given that most of the indicators were partially or completely dependent on VFA concentrations or alkalinity (VFA and alkalinity being interrelated), changes in these two types of process variables could influence the results to a great extent. Regarding the generation of experimental and simulated VFA data points, a major influencing factor was how accurately the model considered the conversion of initial compounds to intermediate and terminal products, compared to reality. This implies that under experimental conditions, the conversion of complex organic substrates to VFA and then further to gases is a function of a series of stochastic and microbial community-driven events, while model simulations, which are relatively simplified descriptions of reality, are generated assuming structured kinetic equations and stoichiometric yield coefficients. Such fundamental differences could inherently lead to deviations between measured and simulated VFA concentrations, despite the good agreement of the experimental and simulated CH_4_ yields (Fig. 1a1, a2) showing that the overall model mass balances are otherwise reliable. On the other hand, the simulation of the various alkalinity fractions posed considerable difficulties. Under laboratory conditions, the alkalinity of a sample is commonly measured through titrimetry, and it is the preferable method for the routine analysis of anaerobic digestion samples as well, due to its speed, simplicity and competitive price [52]. Nonetheless, it involves a significant level of uncertainty when used for the offline measurement of dissolved carbonate concentration in AD samples with inhomogeneous matrices [53]. Meanwhile, as the model cannot simulate titration, simplification was necessary. It was decided that using the simulation results, the BA of the reactor would be calculated by using the bicarbonate and carbonate ion fractions calculated by the model, while IA would be represented by the sum of acetic, propionic and butyric acids, expressed in terms of acetic acid equivalents. However, this simplification meant that any inaccuracies in the simulation of these compounds, together with the uncertainties brought about by the titration method would potentially cause disagreements between experimental and simulated alkalinity results. For this reason, this factor was considered during the evaluation of the comparative results.

Considering the overall sensitivity, stability and measurability of indicators and based on the experimental and simulation results, IA/BA and VFA were selected as optimal early warning indicators. Further to that, IA, BA and VFA/BA were defined as auxiliary indicators for the diagnosis of the AD system treating CS. IA/BA was also suggested as warning indicator in other lab-scale research [32], and an industrial scale research [38], due to its sensitivity to pronounced changes under overloading conditions. VFA was usually recommended as warning indicator [12, 18].

### Threshold value

The abrupt change values and their change amplitude in the previously selected main (IA/BA and VFA) and auxiliary (IA, BA and VFA/BA) early warning indicators were investigated in current study. The variation amplitudes of abrupt changes in indicators monitored during experiments and simulation are shown in Fig. 4, and the accurate values were shown in Tables 2 and 3. The abrupt change value of IA/BA was below 0.7, which was 0.53 for organic gradual overload (R1 g), 0.54 for organic sudden overload (R1 s) and 0.68 for hydraulic gradual overload (R2 g), respectively. Meanwhile, Martín-González et al. [32] proposed the critical value of IA/PA to be 0.24 for municipal waste (37 °C), and in another study Ferrer et al. [38] found this number to be 0.72 for sewage sludge (55 °C). This divergence, however, may be the result of differences in feedstock composition and AD operating conditions. Consequently, compared with providing a determined threshold, observing the relative variation for indicators might be a more promising strategy, while evaluating the effectiveness of early warning indicators. This conclusion found further support in previous studies [12, 18].

Accordingly, an acidification risk that requires attention would appear in present AD processes when the IA/BA changed more than 10% in the experiment or 20% in the simulation, based on the steady-state data.

The abrupt change values of VFA were 0.80 g/L (R1 g), 0.95 g/L (R1 s) and 2.15 g/L (R2 g), respectively. Compared to the average values during the steady-state period, if the VFA increased more than 51% (experimental data) or 19% (simulation data), the biogas system would potentially be at risk from instability.

The abrupt change values of IA, BA and VFA/BA in this study were lower than 0.90, higher than 1.05 and lower than 0.80, respectively. When the IA value increased by nearly 20%, BA value decreased about 11% and VFA/BA value increased approximately 30%. This implied that the biogas system was imbalanced, thus attention should be paid and necessary actions might have to be taken to regulate the AD process.

While based on the assessment of several relevant publications, above discussion could potentially be extended by future analyses of the available literature on the early warning indicators in AD. These, together with experiments carried out and evaluated in a manner similar to the one hereby presented, could offer further model verification, deeper insights into the dynamic behavior of such interconnected processes and eventually provide optimized early warning indicators for biogas plants.

## Conclusion

Monitoring and providing early warning are essential operations in the AD process. Using a mathematical model to simulate the selected experimental process variables provided good data fit and played a key role in the evaluation of the early warning indicators. Based on both experimental and simulated results, the optimal early warning indicators were identified to be IA/BA and VFA. Besides, IA, BA, and VFA/BA could be used as auxiliary indicators for diagnosing the AD system of CS. It is concluded that this modeling can be a promising tool for monitoring the change signals from early warning indicators and improving the standards of AD plant operation.

## Methods

### Feedstock and inoculum

Corn stalk was obtained from Weichang County, Hebei, China. The collected corn stalk was dried and smashed to approximately 3 mm. The inoculum, obtained from an anaerobic reactor in a wastewater treatment plant (Beijing, China), was acclimated at mesophilic temperature by feeding with pig manure for 2 weeks. The properties of corn stalk and inoculum were shown in Table 4.

**Table 4:** The properties of the corn stalk and seeding sludge

Parameters	Unit	Corn stalk	Inoculum
TS	%	90.14 ± 0.32	4.10 ± 0.05
VS	% TS	84.81 ± 0.11	2.20 ± 0.02
Crude fiber	% TS	37.25 ± 0.19	–
Crude protein	% TS	0.49 ± 0.03	–
C	% TS	39.64 ± 0.08	–
N	% TS	0.70 ± 0.02	–
H	% TS	6.38 ± 0.04	–
S	% TS	0.16 ± 0.01	–
C/N	-^a^	56.62 ± 0.45	–
pH	–	–	7.96 ± 0.03

^a^ “–”: undetected

### Experimental setup

The experiment was carried out using two identical 20 L continuously stirred tank reactors (CSTR) having 17 L working volume. The reactors were maintained at mesophilic conditions (35 ± 1 °C) by a heating water bath (SY-200, Changfeng Instrument and Apparatus Company, Beijing, China) and were continuously mixed at a stirring speed of 60 rpm.

Reactor 1 (R1) operated at stepwise elevated OLR by increasing influent feedstock concentration. The experiment in R1 was divided into two phases, which were denoted as gradual overload (day 0–113 with OLR from 1.50 to 2.99 g VS/(L day)) and sudden overload (day 121–165 with OLR from 1.50 to 3.37 g VS/(L day)). Due to process inhibition, the AD system started recovering from day 114 to 120, during which period a re-inoculation was made, substrate feeding was stopped, and the reactor effluent was recycled as feed. R1 was operated at a fixed HRT of 25 days during the whole experiment. At the same time, Reactor 2 (R2) was operated at a gradually increasing OLR, by shortening HRT and keeping influent feedstock concentration at 6% TS. Both reactors were kept operating until the process completely failed. The operational parameters and periods of the experiment are presented in Table 5.

**Table 5:** The operational parameters and duration of the experiment

Reactor no.	Feeding phase	Feed concentration (TS%)	HRT (d)	Duration	OLR (g VS/(L day))
R1	Replacement	4	25	0–25 day	1.50
	Full-load	4	25	26–50 day	1.50
		6	25	51–100 day	2.24
	Gradual overload	8	25	101–113 day	2.99
	Recovery	0	25	114–120 day	0.00
	Sudden overload	4	25	121–128 day	1.50
		6	25	129–137 day	2.24
		8	25	138–146 day	2.99
		9	25	147–165 day	3.37
R2	Replacement	6	30	0–30 day	1.87
	Full-load	6	30	31–60 day	1.87
		6	25	61–110 day	2.24
	Gradual overload	6	20	111–150 day	2.81
		6	15	151–170 day	3.74

Produced biogas was collected in a gas bag, and the gas volume was measured by a gas flowmeter (LML-1, Changchun auto filter co., Ltd, Jilin, China). The effluent was drawn daily for the analysis of pH, VFA, TA, BA, IA, TS and VS.

### Analytical methods

Total solid (TS) and volatile solid (VS) were measured according to the standard methods [54]. Crude protein was estimated by multiplying the total Kjeldahl nitrogen by 6.25 and the total Kjeldahl nitrogen was measured by a Kjeldahl apparatus (K1305A, Sonnen Automated Analysis Instrument Co., Ltd., Shanghai, China). Crude fiber was determined using a fiber analyzer (Model A220, ANKOM Technology Corporation, NY, USA). Organic elemental components of the corn stalk were determined using an elemental analyzer (Exeter Analytical, Inc. CE-440 Elemental Analyzer, Chicago, USA).

Biogas composition (CH_4_, H_2_ and carbon dioxide) was determined by a gas chromatograph (1490, Agilent Technologies, USA) equipped with a thermal conductivity detector as previously described [55]. Liquid samples were centrifuged at 4000 rpm for 10 min and then used for the chemical analyses. Before VFA analysis, samples were filtered through a 0.22 μm membrane. The VFA concentrations were measured by a high performance liquid chromatograph (LC-10A, Shimadzu Corporation, Kyoto, Japan), according to the method proposed by [55]. Alkalinity and pH were tested by an automatic potentiometric titrator (ZDJ-4B, Shanghai INESA Scientific Instrument Co., Ltd, China), with a glass and calomel electrode used as the indicator and reference electrode, respectively. For titration, 0.20 mol/L HCl was used as titrant, and the system was calibrated with anhydrous Na_2_CO_3_. IA, PA, and TA were determined using a three-point method [56], by recording the HCl consumption at the respective pH points of 5.75, 4.3, and 3.8, and converting those values to calcium carbonate (CaCO_3_) equivalents (see Eq. (1)).

1 $${\text{Alkalinity}}\left( {{{{\text{mg CaCO}}_{3} } \mathord{\left/ {\vphantom {{{\text{mg CaCO}}_{3} } {\text{L}}}} \right. \kern-0pt} {\text{L}}}} \right) = \frac{{{\text{HCl concentration}} \times {\text{HCl consumption volume}} \times 50.05}}{\text{Sample volume}} \times 1000,$$

where 50.05 is a coefficient used to convert alkalinity units from mEq/L to mg CaCO_3_/L.BA is estimated by multiplying the PA by 1.25 according to Anderson and Yang [56]. To simplify the data analysis, only BA was analyzed in current study.

### Statistical method

The state of the reactor was defined as steady state when the daily biogas production was within 10% variation, for at least 6 consecutive days [57]. The date of process failure was determined by the time point where a significant decrease (RSD > 20%) appeared in the CH_4_ yield.

Relative standard deviations (RSDs) were calculated according to Eq. 2, for a quantitative assessment of fluctuations in daily indicator values compared with the previous day.

2 $${\text{RSD}} = \frac{S}{{\overline{x} }} \times 100\% = \frac{{\sqrt {\mathop \sum \nolimits_{i = 1}^{2} (x_{i} - \overline{x} )^{2} } }}{{\overline{x} }} \times 100\%$$

In the above equation, *S* is the standard deviation of the measured indicator value compared to the previous day, and $\overline{x}$ is the average of the values at day i and i−1. The larger the RSD value, the greater the fluctuation. In the current study, RSD > 10% (yellow symbol in all figures) and RSD > 20% (red symbol in all figures) were identified as the signs of slightly and highly unstable process, respectively. Thereby, the date when RSD exceeds 20% was determined as the time point where sudden changes took place.

For the quantification of goodness of fit between simulations and experimental data, relative root mean-squared error (rRMSE) and mean absolute percentage error (MAPE) were used, according to Eqs. 3 and 4.

3 $${\text{rRMSE}} = \frac{1}{{\overline{y}_{exp} }}\sqrt {\frac{1}{n}\mathop \sum \limits_{i = 1}^{n} \left( {y_{{exp_{i} }} - y_{{sim_{i} }} } \right)^{2} }$$

4 $${\text{MAPE}} = \frac{100}{n}\mathop \sum \limits_{i = 1}^{n} \left| {\frac{{y_{{exp_{i} }} - y_{{sim_{i} }} }}{{y_{{exp_{i} }} }}} \right|,$$

where $y_{{sim_{i} }}$ and $y_{{exp_{i} }}$, respectively, represent a single simulated or measured (experimental) data value, while $\overline{{y_{\exp } }}$ is the average of all experimental data values and $n$ is the total number of experimental data points available. RMSE is commonly used for AD model evaluation [58, 59] and in certain cases, offers advantages over other measures, especially when sensitivity to large errors between experimental and simulated data points is required [60]. After dividing RMSE by the mean of the experimental data points involved, the resulting rRMSE can be compared through different variable datasets, without having to consider its specific units. While rRMSE is defined in the range of 0 (no error) to infinity (no fit) and can therefore take significantly different values for different variables, MAPE is expressed in percentages. By design, it takes values between 0 and 100% for simulated data points that are at most double as large as experimental ones, but is in general said to produce reliable simulations when MAPE is less than 50% [61]. Consequently, it offers a complementary metric for error assessment less distorted by extreme data points, and provides a holistic view of dynamic simulation regression [62].

#### Mathematical model simulations

For the simulation of the two experimental reactors and the different overload conditions, an advanced bioconversion model (BioModel) was used. The model was developed by Angelidaki et al. [63, 64], and was later extended by Kovalovszki et al. [65] and Lovato et al. [66], considering various anaerobic co-digestion scenarios for validation. Compared to the extended model established earlier, however, two minor changes were made in the present model implementation. Of the two, the first involved the removal of ammonia as a microbial growth limiting substrate from the model’s kinetic equations, while through the second, the acetic acid inhibition effect on acetolactic methanogens was replaced by a total VFA inhibition effect. Former change was considered reasonable, given that the single substrate in these experiments—corn stalk—is a negligible source of ammonia. Meanwhile, the argument for extending the range of VFA inhibition on methanogens lies in their sensitivity to undissociated organic acids [67], as well as the dilute reactor medium, which in general might limit the availability of physical shelter and granule-forming sites for methanogens [68, 69]. The above described VFA inhibition effect was kinetically controlled similar to the original acetic acid inhibition effect, using inhibition constants with manually estimated values of 315 mg/L and 365 mg/L for R1 and R2, respectively. By comparison, similar inhibition constants found in published literature take values on a wide range, from 10 s to 1000 s mg/L and considering either acetic and propionic acid as the main inhibitors, or all VFA collectively [64, 70–73]. The values reported depend largely on the specific reactor conditions and substrates, therefore the low values used in this work can be justified by the highly dilute reactor contents and the lack of solid buffers (potentially leading to faster acidification) and the general sensitivity of methanogenic archaea to the accumulation of acids. In addition, the slight difference in the magnitude of the values estimated appears negligible compared to the above literature sources, and can be attributed to reactor-specific conditions.

## Additional files

**Additional file 1: Fig. S1.** Variation of effluent TS and VS in (a) R1 and (b) R2. **Fig. S2.** Variation of pH in (a) R1 and (b) R2.

## Abbreviations

AD: anaerobic digestion
BA: bicarbonate alkalinity
BioModel: bioconversion model
CS: corn stalk
CSTR: continuously stirred tank reactors
HRT: hydraulic retention time
IA: intermediate alkalinity
OLR: organic loading rates
R: reactor
RSD: relative standard deviations
TA: total alkalinity
TS: total solid
VFA: volatile fatty acid
VS: volatile solid
