# Supplementary material for: Early warning indicators for mesophilic anaerobic digestion of corn stalk: a combined experimental and simulation approach
Source: Biotechnol Biofuels. 2019 May 3;12:106. doi: 10.1186/s13068-019-1442-7 (PMC6498497; doi:10.1186/s13068-019-1442-7)
Supplement: Supplementary file 1 — Additional file 1: Fig. S1. Variation of effluent TS and VS in (a) R1 and (b) R2. Fig. S2. Variation of pH in (a) R1 and (b) R2. [file 13068_2019_1442_MOESM1_ESM.docx]

Early warning indicators for mesophilic anaerobic digestion of corn stalk: a combined experimental and simulation approach

Yiran Wu et al. Additional Information

**Fig. S1**  Variation of pH in (a) R1 and (b) R2.

**
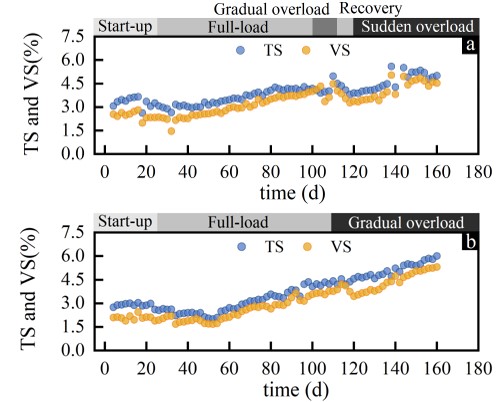
**

**Fig. S2** Variation of pH in (a) R1 and (b) R2.

**
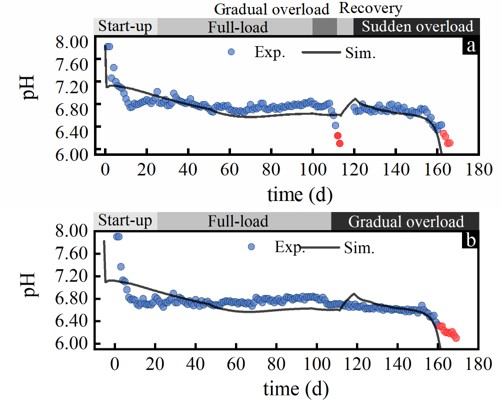
**
